# First Complete Genome Sequences of Dengue Virus Serotype 2 Strains from the Solomon Islands and Vanuatu

**DOI:** 10.1128/MRA.01360-18

**Published:** 2019-01-10

**Authors:** Alyssa T. Pyke, Jane Cameron, Jamie McMahon, Amanda De Jong, Peter Burtonclay

**Affiliations:** a Public Health Virology Laboratory, Forensic and Scientific Services, Coopers Plains, Queensland, Australia; University of Rochester School of Medicine and Dentistry

## Abstract

Isolates of dengue virus serotype 2 (DENV-2) were recovered from a female resident of the Solomon Islands in 2016 and another female patient who had traveled from Vanuatu to Australia in 2017. Here, we describe the first complete genome sequences of DENV-2 strains from Vanuatu and the Solomon Islands.

## ANNOUNCEMENT

Dengue virus serotype 2 (DENV-2; genus Flavivirus, family Flaviviridae) (1) has recently caused several outbreaks of dengue disease in the Pacific region, including the Solomon Islands and Vanuatu (2–4). Between August 2016 and April 2017, 12,250 dengue virus cases with 10 hospitalizations were recorded in the Solomon Islands, and in Vanuatu, 2,838 cases with 147 hospitalizations were reported between November 2016 and May 2017 (2).

In October 2016, a female in her 70s residing in the Solomon Islands presented with headache, fever, and body aches. In January 2017, a second female patient in her 20s developed symptoms of diarrhea and joint pain after traveling from Vanuatu to Brisbane, Australia. DENV-2 RNA was detected in acute-phase serum from both patients by reverse transcription real-time PCR (RT-rtPCR) (5, 6), and DENV-2 isolates, namely, SI 2016 and Van 2017, were subsequently recovered from each serum sample.

Here, we describe the whole-genome sequencing (WGS) of SI 2016 and Van 2017 and the first report of complete DENV-2 genome sequences from isolates obtained from the Solomon Islands and Vanuatu. Briefly, DENV-2 RNA was extracted from the SI 2016 and Van 2017 isolates (C6/36 cell culture supernatant, passage 1) using the QIAamp viral RNA extraction kit (Qiagen, Chadstone, Australia). The extracted viral RNAs were circularized to enable complete sequencing of the terminal 5′ and 3′ untranslated regions using a tobacco decapping enzyme (Enzymax LLC, USA) and T4 RNA ligase (New England Biolabs, Genesearch, Arundel, Australia) as reported previously (7). To remove host and potentially contaminating microbial DNA, the circularized RNA was then DNase treated (Heat & Run kit, ArcticZymes, Scientifix, South Yarra, Australia). Whole-genome sequencing was performed on a NextSeq 500 instrument (Illumina, San Diego, CA) as described previously (7, 8) using the Nextera XT kit for cDNA library construction, followed by paired-end (2 × 151-nucleotide [nt]) sequencing using the V2 mid output kit. Ethical approval for this study was granted by the Forensic and Scientific Services Human Ethics Committee.

Illumina sequencing yielded 15,050,786 and 10,303,566 for SI 2016 and Van 2017, respectively, and raw sequence reads were processed using Geneious R10 (version 10.2.3) software (9). Low-quality reads were removed using BBDuk (version 37.25; minimum quality score, 20). Trimmed reads were assembled by mapping to a reference genome (GenBank accession number AB189124) with complete 5′ and 3′ untranslated terminal sequences using default parameters and the low-sensitivity setting. To compare the SI 2016 and Van 2017 genome sequences with all other available DENV-2 sequences on GenBank, BLASTN analyses were performed using an expect threshold of 10. For SI 2016, a total of 3,861,001 (from 5,400,568) reads mapped to the reference genome (AB189124), with an average coverage depth of 48,014×. The SI 2016 complete genome (10,723 nt; G+C content, 45.7%) and envelope (E) gene (1,485 nt) sequences demonstrated 97% nt sequence identity with the reference genome and 99% nt sequence identities with DENV-2 strains from Papua New Guinea in 2013 (KU517845) and the Solomon Islands in 2016 (KY495808). For Van 2017, a total of 1,916,389 (from 3,144,522) reads mapped to the reference genome, with an average coverage depth of 23,840×. The Van 2017 complete genome (10,722 nt; G+C content, 46.2%) and E gene (1,485 nt) sequences demonstrated 98% nt sequence identity with the reference genome and 98% and 100% nt sequence identities with DENV-2 strains from China in 2001 (KR920365) and French Polynesia in 2017 (from a patient who had traveled to Vanuatu; KY782126), respectively. The complete genomes of S1 2016 and Van 2017 (both belonging to the DENV-2 Cosmopolitan genotype) share only 96% nt sequence identity, highlighting the potential diversity of DENV-2 strains responsible for concurrent outbreaks in the Pacific. These data are important for the ongoing surveillance and monitoring of DENV strains which continue to be a major public health threat throughout the Pacific and wider global regions in which Aedes mosquito vectors are prevalent.

### Data availability.

Raw sequencing reads were deposited in NCBI under the BioProject accession number PRJNA493303 (SRA numbers SRX4776970 and SRX4776971). The consensus genome sequences have been deposited in GenBank under accession numbers MH985858 (SI 2016) and MH985859 (Van 2017).

## References

[1] Calisher CH, Karabatsos N 1988 Arbovirus serogroups: definition and geographic distribution, p 19–57. *In* Monath TP (ed), The arboviruses: epidemiology and ecology, vol I CRC Press, Inc, Boca Raton, FL.

[2] ReliefWeb. 2016 Pacific: Dengue Outbreak—October, 2016. United Nations Office for the Coordination of Humanitarian Affairs, New York, NY https://reliefweb.int/disaster/ep-2016-000112-slb.

[3] Aubry M, Teissier Y, Mapotoeke M, Teissier A, Giard M, Musso D, Cao-Lormeau VM 2017 High risk of dengue type 2 outbreak in French Polynesia, 2017. Euro Surveill 22. doi:10.2807/1560-7917.ES.2017.22.14.30505. PMC5388125 28422007

[4] Moore PR, van den Hurk AF, Mackenzie JS, Pyke AT 2017 Dengue viruses in Papua New Guinea: evidence of endemicity and phylogenetic variation, including the evolution of new genetic lineages. Emerg Microbes Infect 6:e114. doi:10.1038/emi.2017.103. 29259329 PMC5750459

[5] Pyke AT, Gunn W, Taylor C, Mackay IM, McMahon J, Jelley L, Waite B, May F 2018 On the home front: specialised reference testing for dengue in the Australasian region. Trop Med Infect Dis 3:75. doi:10.3390/tropicalmed3030075. PMC6161173 30274471

[6] Callahan JD, Wu SJ, Dion-Schultz A, Mangold BE, Peruski LF, Watts DM, Porter KR, Murphy GR, Suharyono W, King CC, Hayes CG, Temenak JJ 2001 Development and evaluation of serotype- and group-specific fluorogenic reverse transcriptase PCR (TaqMan) assays for dengue virus. J Clin Microbiol 39:4119–4124. doi:10.1128/JCM.39.11.4119-4124.2001. 11682539 PMC88496

[7] Pyke AT, Huang B, Warrilow D, Moore PR, McMahon J, Harrower B 2017 Complete genome sequence of a highly divergent dengue virus type 2 strain, imported into Australia from Sabah, Malaysia. Genome Announc 5:e00546-17. doi:10.1128/genomeA.00546-17. 28729258 PMC5522925

[8] Huang B, Pyke AT, McMahon J, Warrilow D 2017 Complete coding sequence of a case of chikungunya virus imported into Australia. Genome Announc 5:e00310-17. doi:10.1128/genomeA.00310-17. 28495775 PMC5427210

[9] Kearse M, Moir R, Wilson A, Stones-Havas S, Cheung M, Sturrock S, Buxton S, Cooper A, Markowitz S, Duran C, Thierer T, Ashton B, Meintjes P, Drummond A 2012 Geneious Basic: an integrated and extendable desktop software platform for the organization and analysis of sequence data. Bioinformatics 28:1647–1649. doi:10.1093/bioinformatics/bts199. 22543367 PMC3371832

